# Nanoparticle-Based mRNA Vaccine Induces Protective Neutralizing Antibodies Against Infectious Bronchitis Virus in In-Vivo Infection

**DOI:** 10.3390/vaccines13060568

**Published:** 2025-05-26

**Authors:** Aseno Sakhrie, Ankarao Kalluri, Zeinab H. Helal, Challa V. Kumar, Mazhar I. Khan

**Affiliations:** 1 Department of Pathobiology, University of Connecticut, Storrs, CT 06269, USA; aseno.sakhrie@uconn.edu (A.S.); zeinab.helal@uconn.edu (Z.H.H.); 2 Department of Material Science, University of Connecticut, Storrs, CT 06269, USA; ankarao.kalluri@uconn.edu (A.K.); challa.kumar@uconn.edu (C.V.K.); 3 Connecticut Veterinary Medical Diagnostic Laboratory, Department of Pathobiology, University of Connecticut, Storrs, CT 06269, USA

**Keywords:** nanoparticles, mRNA vaccine, infectious bronchitis virus

## Abstract

**Background:** Live attenuated and inactivated virus vaccines are commonly used against infectious bronchitis virus (IBV) in chickens, but they have limitations such as mutation risks and short efficacy. This study explores cationic bovine serum albumin (BSA) polyamine nanoparticles (NPs) for delivering IBV spike protein mRNA, aiming to develop a safer and more effective vaccine. **Methods**: A BSA-based nanoparticle system was designed with positive surface charges and characterized using dynamic light scattering (DLS), Zetasizer, and transmission electron microscopy (TEM). Its cytotoxicity, cellular uptake, and ability to deliver IBV spike protein mRNA were evaluated in macrophage-like chicken cell lines (HD11), followed by immunogenicity studies in SPF chickens to assess immune responses. **Results**: The study demonstrated successful binding and transfection efficiency of the in vitro transcription (IVT)-mRNA complexed with the NPs, which was enhanced with chloroquine. Immunogenicity studies in SPF chickens showed a significant increase in antibody titers in chickens vaccinated with the mRNA vaccine compared to the PBS control, indicating an effective immune response against the IBV S protein. Furthermore, the neutralization index doubled after a higher-dose mRNA booster with chloroquine, and PBMCs from immunized chickens exhibited a threefold higher stimulation index than the PBS control. **Conclusions**: BSA-based NPs effectively deliver IBV spike protein mRNA, enhancing immune responses and offering a promising strategy for a safer, more effective IBV vaccine.

## 1. Introduction

Infectious bronchitis virus (IBV) is a highly contagious avian coronavirus that affects chickens of all ages, leading to severe economic losses in the poultry industry by reducing weight gain and egg production [1]. Symptoms include respiratory distress, reproductive system defects, and nephritis. Vaccination is the primary control strategy, with live attenuated vaccines used in broilers and inactivated vaccines for layers. However, live attenuated vaccines pose risks such as reversion to virulence, secondary bacterial infections, and recombination with virulent strains [2,3]. Inactivated vaccines require priming, multiple doses, and adjuvants to enhance immunogenicity [4].

IBV is caused by a virus belonging to the family *Coronaviridae* and order *Nidovirales* [5]. Coronaviruses have been divided into three groups, based on serologic relationships and phylogenetic clustering- the alpha-, beta-, and gammacoronaviruses. IBV belongs to gammacoronavirus. It is an enveloped virus with a positive sense single-stranded RNA with a genome size of 27.6 kb. It consists of a large replicase gene that encodes 15 non-structural proteins (nsps); the structural protein includes the spike protein S, the envelope protein E, the membrane protein M and the nucleocapsid protein N; and the accessory genes including 3a, 3b, 4b, 4c, 5a, and 5b [6]. Spike protein serves as an important focus for vaccine development because it is the primary antigenic component that is responsible for inducing neutralizing antibodies and establishing protective immunity against virus infection. The S protein facilitates the attachment of the virus to specific receptors on the surface of host cells. It initiates the merging of the viral envelope with the host membrane, allowing the viral genetic material to enter the host cell [7]. The receptor binding sites in the S protein was mapped in the 223-residue region (aa 506–729) of the S1 subunit for the first time in transmissible gastroenteritis virus (TGEV) of pigs, which is an alphacoronavirus [7]. In the study, the neutralizing monoclonal antibody was able to block the 223-residue polypeptide from binding to the receptor. In IBV virus as well, the S1 protein bears the receptor binding domain, which is responsible for virus attachment to host cells [8] and induces neutralizing antibodies [9].

While advancements in viral vector-based and DNA vaccines have shown some promise in improving immune responses, they are not without challenges. For example, recombinant fowlpox viruses expressing the IBV S1 gene have been shown to reduce issues related to RNA mutation but suffer from pre-existing immunity to the vector and protein misfolding [10,11,12,13]. Similarly, subunit vaccines utilizing the S1 or N proteins of IBV aim to induce neutralizing antibodies and CTL responses but still face limitations in terms of efficacy and stability [9,14,15].

DNA vaccines have shown strong immune responses, especially when combined with live attenuated vaccines in boosting protocols. However, the challenges of large-scale administration and the potential for poor immunogenicity in some cases remain obstacles to their widespread use [16,17,18,19,20,21]. Reverse genetics approaches have enabled the modification of IBV genomes, replacing virulent S glycoprotein genes with attenuated ones to develop vaccines [22,23]. Recent advancements in BAC-based reverse genetics systems have further refined IBV vaccine development [24]. Despite its potential, biosafety concerns and viral sequence recovery remain limitations.

Given these challenges, there is a clear need for novel vaccine technologies that can overcome the limitations of current strategies. We propose nanoparticle (NP)-based mRNA vaccines as a promising alternative. These vaccines offer several advantages over traditional approaches, including improved stability, enhanced immune responses, and the ability to deliver mRNA in a controlled, targeted manner. Specifically, bovine serum albumin (BSA)-based NPs are an ideal platform due to their low toxicity, biodegradability, and effectiveness in both drug delivery and vaccine applications [25,26,27,28,29,30,31]. This platform can be modified to carry different functional groups, enhancing their suitability for vaccine delivery. The continuous threat of IBV recombination between virulent strains underscores the need for innovative vaccine development that can provide rapid adaptation to emerging strains. The flexibility of mRNA vaccines, combined with the precise delivery capabilities of NP technology, allows for more adaptable vaccine strategies that can be quickly modified to address new variants of IBV.

In our study, we designed an IBV mRNA vaccine utilizing an in vitro transcribed (IVT) S protein delivered via bovine serum albumin pentaethylenehexamine (BSA-PEHA) NPs. We evaluated NP cytotoxicity, cellular uptake kinetics, mRNA interaction, and the immunogenic potential of the nanocomplex. Also, we utilized chloroquine (CHQ) in conjunction with the BSA-PEHA NPs to further enhance the delivery and efficacy of the mRNA vaccine. CHQ is known to increase endosomal pH, which aids in the endosomal escape of mRNA, thereby facilitating its release into the cytosol and enhancing transfection efficiency.

## 2. Materials and Methods

### 2.1. Cationic BSA NP Synthesis and Characterization

A 1 M pentaethylenehexamine (Sigma Aldrich, Burlington, MA, USA) solution was prepared by dissolving the amine in an equal amount of water and adjusting the pH to 5.5 with concentrated HCl while keeping it on ice. The solution was then diluted to the desired volume with deionized water (DI H_2_O). Separately, 100 mg of BSA was dissolved in 4 mL of DI H_2_O and stirred for 1 h. Next, 5.37 mL of the 1 M amine solution was added and stirred for 45 min; then, 1 mL from 1 M [N-(3-Dimethylaminopropyl)-N′-ethyl carbodiimide hydrochloride (EDC) solution was added. The reaction proceeded under nitrogen for 4 h before dialysis into a 10 mM NaH_2_PO_4_ buffer at pH 7.0. The NPs were characterized using transmission electron microscopy (TEM-FEI Tecnai T12 S/TEM, FEI, Hillsboro, OR, USA), dynamic light scattering (DLS-CoolBatch+, Precision Detectors, Amherst, MA, USA), and zeta potential analyzer.

### 2.2. NP Uptake Assay Using HD11 Cells

The protocol from Da silva et al. 2018 was modified and used for our study [32]. HD11 cells (provided by Dr. Silbart Lawrence) were cultured in 35 mm glass-bottom dishes with RPMI 1640 medium (Gibco, Thermo Fisher Scientific, Waltham, MA, USA) containing 10% FBS (Gibco, Thermo Fisher scientific, Waltham, MA, USA) and 1% Antibiotic-Antimycotic (Gibco, Thermo Fisher scientific, Waltham, MA, USA) at 42 °C for 24 h. Cells were then treated with FITC-labeled BSA-PEHA NPs (10 µg/mL) and incubated for 0.5, 1, and 24 h. After incubation, NPs were rinsed with PBS, and cells were stained with CellMask™ Deep Red Plasma Membrane stain (Thermo Fisher scientific, Waltham, MA, USA) for 10 min at 42 °C. The staining solution was removed, and Hoechst 33,342 was added, followed by a 5–10-min incubation in the dark. Live-cell imaging was carried out on a Nikon A1R spectral confocal microscope equipped with a 60× objective lens. Fluorescence detection was performed using excitation/emission settings of 460/490 nm for FITC and 649/666 nm for CellMask™ Deep Red plasma membrane stain.

### 2.3. In Vitro Cytotoxicity of BSA-PEHA NPs Determined Using an MTT Assay

The cytotoxic effects of BSA-PEHA nanoparticles were assessed in vitro using the TACS MTT Cell Proliferation Assay (R&D Systems, Minneapolis, MN, USA), following the protocol provided by the manufacturer, and employing HD11 cells. In summary, cells were plated in 96-well culture plates at a density of 5000 cells per well and incubated overnight at 42 °C in 0.1 mL of culture medium composed of 90% RPMI, 10% fetal calf serum, and 1% antibiotic-antimycotic. Following incubation, cells were exposed to nanoparticles at concentrations ranging from 100 µg/mL to 0.097 µg/mL. The cells were then incubated for 24, 48 and 72 h. After each incubation, the MTT reagent was added to all the wells and kept for 4 h at 37 °C. 100 µL of the detergent reagent was finally added and the cells were kept at RT overnight. After the incubation, the absorbance in each well was read at 570 nm using a microplate reader (Molecular Devices SpectraMax Plus 384, Molecular Devices, San Jose, CA, USA). The controls were cell culture medium, BSA, and PEHA.

#### In Vitro Cytotoxicity Study Using Flow Cytometry

The cells were stained using a commercially available kit, Viability/Cytotoxicity Assay Kit (Biotium Inc., Hayward, CA, USA) for live non-viable cells were identified in accordance with the guidelines provided by the manufacturer. HD11 cells were plated in 12-well plates at a seeding density of 2–2.5 × 10⁴ cells per cm^2^ and allowed to adhere and grow for 24 h. Subsequently, the cells were exposed to BSA-PEHA nanoparticles at concentrations ranging from 200 µg/mL to 1 µg/mL and incubated for an additional 24-h period. After washing with serum-free media, cells were stained with a solution containing 4 µM calcein AM and 4 µM EthD-III for 30–45 min at room temperature. Flow cytometry analysis was conducted using a BD FACSymphony A5 SE (BD Biosciences, San Jose, CA, USA) to detect calcein in the FITC channel and EthD-III in the PE channel.

### 2.4. Electrophoretic Mobility Shift Assay (EMSA)

A horizontal electrophoresis device (Sub-cell GT, Bio-Rad, Hercules, CA, USA) was used for agarose gel electrophoresis with a 0.5% agarose gel prepared in 40 mM tris-acetate buffer (pH 7.2). Ethidium bromide (0.5 µg/mL) was added for eGFP mRNA detection. BSA-PEHA NPs (25 µg/mL) were conjugated with 1 µg and 2 µg of mRNA and incubated for 10 min. Samples were mixed with 50% loading buffer (glycerol and bromophenol blue) before electrophoresis at 100 V, 120 mA for 25 min alongside controls (BSA, PEHA, and BSA-PEHA). The gel was stained overnight with Coomassie blue in 10% acetic acid and de-stained overnight in 10% acetic acid.

### 2.5. Circular Dichroism

Structural changes resulting from the binding of mRNA with BSA-PEHA NPs were evaluated using a CD spectroscopy instrument Jasco J-710 CD spectrophotometer (JASCO, Easton, MD, USA). Following a 5 min equilibration period at 25 °C, the BSA-PEHA secondary structure and DNA maximum and minimum changes were observed using a wavelength range of 200–300 nm. The bandwidth and sensitivity were set at 1 nm and 20 mdeg, respectively, and step resolution was maintained at 1 nm/data point. Averaging three scans at a speed of 100 nm/min was used. A 10 mm quartz cuvette was used to collect the spectra, and the data were normalized to the DNA or protein’s micromolar concentration per unit route length. After deducting the blank, the samples’ final spectra were obtained by mixing different concentrations of BSA-PEHA NPs and keeping the concentration of the mRNA constant, and also keeping the BSA-PEHA NPs constant and increasing the mRNA concentrations.

### 2.6. Transfection of Cells with Lipofectamine 2000 and BSA-PEHA NPs

For transfection studies, HD11 cells were plated in 6-well culture dishes at a density of 3 × 10^5^ cells per well in RPMI 1640 medium supplemented with 10% FBS and 1% Antibiotic-Antimycotic. The cells were incubated at 42 °C for 24 h. Following incubation, the medium was aspirated, and the cells were rinsed with DPBS. The transfection procedure followed the method described by Avci-Adali et al. (2014) [33]. Briefly, 2.5 µg of eGFP mRNA and 2 µL of Lipofectamine 2000 were mixed with 100 µL of Opti-MEM I reduced serum medium and maintained at room temperature for 20 min before being added to the cell monolayer. In a separate tube, 100 µL of 25 µg/mL BSA-PEHA was mixed with 2.5 µg of eGFP mRNA, incubated for 10 min, and then added dropwise to the cell monolayer. 500 µL of Opti-MEM I reduced serum medium was added to each well, and the cells were incubated at 42 °C for 4 h. Following incubation, the transfection medium was replaced with fresh RPMI 1640, and after 24 h, fluorescence microscopy was used to assess transfection efficiency.

### 2.7. Design of IVT S Protein mRNA for IBV and Transfection in HD11 Cells

Custom-made mRNA was utilized for this study to suit the experimental requirements. The mRNA was synthesized by Cellerna Bioscience (Baesweiler, Germany) using a custom synthesis service. The open reading frame corresponding to the target antigen, the S protein, was located using the BLAST^®^ tool available through the National Center for Biotechnology Information (NCBI).

The accession number AY851295 was selected. The mRNA sequence has a length of 3489 nucleotides (Supplementary Figure S4), encoding for spike protein, and was fully substituted with 5-methoxyuridine to enhance stability and reduce immunogenicity. Cap1 analog and a 110 bp poly A-tail were incorporated into the mRNA synthesis process and a purity of over 98% was ensured.

The HD 11 cells were grown on a 6-well plate as previously described. 100 µL of 25 µg/mL BSA-PEHA NPs and 2.5 µg of IVT S protein mRNA were mixed, incubated for 10 min, and added dropwise to the cell monolayer. 500 µL of Opti-MEM I reduced serum media containing 25 mM of chloroquine diphosphate (Sigma-Aldrich, Burlington, MA, USA) was then added to the transfected wells and incubated for 4 h at 42 °C. After the incubation, the transfection media was replaced by RPMI 1640 with 25 µM of chloroquine and then incubated for 24 h at 42 °C.

#### 2.7.1. SDS PAGE

Twenty-four hours post-transfection, the culture medium was discarded, and cells were rinsed twice with ice-cold DPBS. Each well of the 6-well plate was then treated with 100 µL of chilled RIPA lysis buffer and incubated on ice for 5 min with occasional gentle agitation. Cell lysates were subsequently collected and centrifuged at approximately 14,000× *g* for 15 min, after which the supernatant was retained for downstream analysis. The collected samples were combined with 2× Laemmli sample buffer supplemented with 2-mercaptoethanol, heated at 95 °C for 5 min, allowed to cool, and stored at −20 °C until further use.

Then, 15 µL of each sample was loaded onto a 10% Mini-PROTEAN^®^ TGX™ gel (BioRad, Hercules, CA, USA) and run for 1 h at 120 V, 60 mA in tris/glycine buffer. The gel was stained with Coomassie stain 1 (0.003% Coomassie, 10% acetic acid, 10% isopropanol) for 30 min, followed by stain 2 (0.003% Coomassie, 10% acetic acid) until bands appeared. Destaining was performed overnight in 10% acetic acid, and imaging was done using a Bio-Rad ChemiDoc MP System (BioRad, Hercules, CA, USA).

#### 2.7.2. Western Blotting

After SDS-PAGE, proteins were transferred to a nitrocellulose membrane by placing the gel holder cassettes into a Mini-Trans-Blot Cell (BioRad, Hercules, CA, USA). A transfer sandwich was assembled in the following order: sponge, filter paper, nitrocellulose membrane, gel, filter paper, and sponge. The transfer was carried out at 20 V, 100 mA for 2.5 h on ice to maintain the temperature at 4 °C. Following the transfer, the membrane was blocked with 5% BSA in PBST for 1 h at room temperature.

After that, primary antibody Chicken polyclonal (pAb): anti-Infectious Bronchitis (Merck Animal Health, Boxborough, MA, USA) antibody (ab31671) (Abcam, Waltham, Boston, USA) (1:10,000) and housekeeping protein Rabbit mAb GAPDH (1:1000) (Abcam, Waltham, Boston, MA, USA) was added. Nitrocellulose paper was rinsed 3 times for 5 min each with PBST. A secondary antibody-HRP-conjugated Rabbit anti-Chicken IgY (IgG) (H + L (1:10,000) (Abclonal, Woburn, MA, USA) for IBV and HRP Goat Anti-rabbit IgG (H + L) (1:10,000) (Abclonal, Woburn, MA, USA) for housekeeping protein was added and incubated for 1 h at room temperature. The membrane was rinsed again 3 times for 5 min each with PBST. The substrate working solution was prepared with equal amounts of substrate and stable peroxide components of the SuperSignal™ West Atto Ultimate Sensitivity Chemiluminescent Substrate (Thermo Fisher Scientific, Waltham, MA, USA). Sufficient amounts of the substrate mixture were added to the membrane and incubated for 5 min. The image of the membrane was taken in BioRad ChemiDoc MP Systems (BioRad, Hercules, CA, USA).

### 2.8. Animal Experiment

Four-week-old SPF white leghorn chickens (*n* = 6 chickens) were inoculated via an intramuscular route with two doses (5 µg and 25 µg-with and without chloroquine) of IVT S protein IBV mRNA complexed with the BSA-PEHA NP. A PBS control group was also included. After determining the cytotoxicity of chloroquine in vitro (Supplementary Figure S3), a concentration of 25 µM was chosen to be used for the study. A booster dose of the vaccine was administered after 2 weeks of the first vaccination. The sera from the chickens were collected at 14 and 28 days of inoculation. At the end of the study, the chickens were euthanized following the procedure approved by the University of Connecticut Institutional Animal Care and Use Committee ((IACUC protocol no. A21-037).

#### 2.8.1. Enzyme-Linked Immunosorbent Assay (ELISA)

ELISA was performed using the IDEXX IBV Ab test kit (Idexx, Westbrook, ME, USA), following the manufacturer’s protocol.

#### 2.8.2. Lymphocyte Proliferation

PBMCs were isolated from chicken whole blood using Histopaque-1077 (Sigma Aldrich, Burlington, MA, USA) gradient centrifugation, resuspended, stained with trypan blue, and counted [34]. The concentration of PBMCs was adjusted to 10^7^ cells/mL. Subsequently, 100 µL of PBMCs from each chicken, at a concentration of 1 × 10^6^ cells/well, then cells were seeded into 96-well plates and stimulated with Concanavalin A and heat-inactivated IBV M41 virus. After 48 h of incubation at 41 °C with 5% CO₂, cell proliferation was assessed using the Roche BrdU colorimetric ELISA kit (Millipore Sigma Burlington, MA, USA) with BrdU incorporation measured at 450 nm. The Stimulation Index (SI) was calculated by comparing OD values of antigen-stimulated and medium-treated PBMCs using the following formula:

$$\mathrm{SI}=\frac{OD\;value\;\left(antigen-stimulated\;PBMCs\right)}{OD\;value\;\left(medium\;treated\;PBMCs\right)}$$

#### 2.8.3. Virus Neutralization

The virus neutralization study was done using the alpha method, as described by WOAH, 2018 [35]. Briefly, the sera were separated from the blood collected from the vaccinated chickens and inactivated by heat at 56 °C for 30 min. A ten-fold dilution (up to 6 dilutions) was made with the IBV M41 virus (10^−7^) and mixed with the inactivated antiserum (0.5 mL) and incubated for 30 min at room temperature. The mixture was then inoculated into an allantoic sac of 9–11-day-old embryonated specific pathogen-free (SPF) chicken eggs (*n* = 5). The inoculated eggs were incubated for 3–4 days. The endpoints were calculated using the Reed and Muench method. The results are expressed as a neutralization index (NI) representing the log10 difference in the titer of the virus alone and that of the virus/antiserum mixtures. The NI values of <1.5 are not specific.

#### 2.8.4. Statistical Analysis

Data were analyzed using one or two-way ANOVA with Tukey’s multiple comparison test in GraphPad prism 10.0 (GraphPad Software, San Diego, CA, USA). Experiments were independently repeated three times at least in triplicate for the in vitro studies. Statistical significance was achieved at levels of *p* values < 0.05 (*), <0.01 (**), and <0.001 (***).

## 3. Results

### 3.1. BSA-PEHA NP Synthesis and Characterization

**Figure 1:**
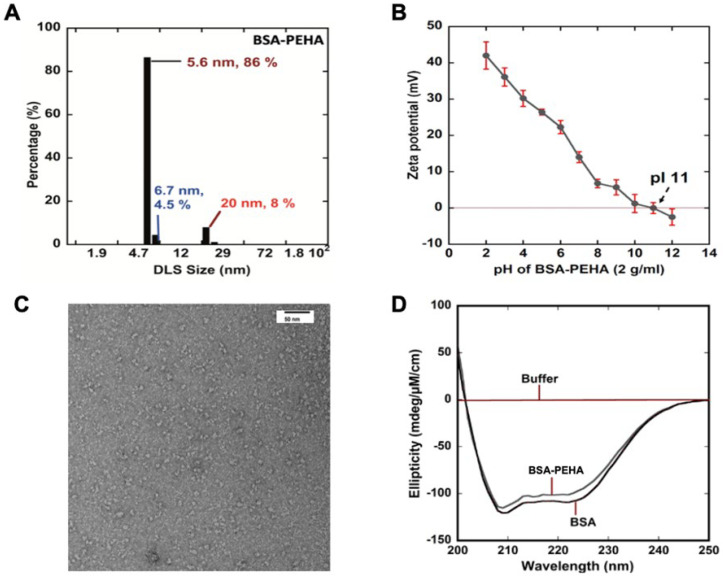
Characterization of BSA-PEHA NPs. (**A**) Dynamic Light Scattering of BSA-PEHA (10 mM phosphate buffer pH 7.0); (**B**) Zeta potential; (**C**) TEM image of BSA-PEHA (pH 4.0); (**D**) Circular Dichroism shows no loss in ellipticity for BSA-PEHA (blue) compared to BSA (red). All samples show a double minimum at 208 and 222 nm.

DLS analysis revealed peaks corresponding to particle diameters of 5.6 nm (86%), 6.7 nm (4.5%), and 20 nm (8%) in a 40 mM phosphate buffer at pH 7.2 (Figure 1A). The DLS data were further validated by transmission electron microscopy (TEM) images (Figure 1C), which showed particles approximately 10 nm in diameter. To assess the secondary structure of BSA within the particles, CD spectroscopy was performed. The far-UV CD spectrum of BSA-PEHA (in 40 mM phosphate buffer, pH 7.2) displayed minima at 222 and 208 nm (Figure 1D), closely matching the spectrum of unmodified BSA. The modification of BSA with PEHA caused minimal alterations to the CD spectrum, suggesting that the crosslinking process had little to no impact on the protein’s structure. Thus, the protein structure remained largely unchanged upon modification. Zeta potential measurements were conducted to evaluate changes in the surface charge of the nanoparticles. As shown in Figure 1B, pH levels significantly affected the zeta potential, with the particles acquiring a more positive charge in acidic conditions. Generally, colloidal systems with zeta potential values in the range of ±20–30 mV or higher are considered stable.

### 3.2. Cellular Uptake of BSA-PEHA by Chicken Macrophage-like HD 11 Cells

**Figure 2:**
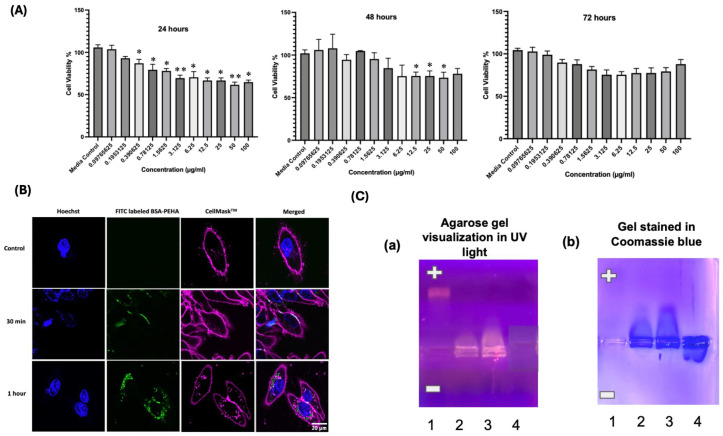
(**A**) Cell viability of BSA-PEHA NPs treated HD11 cells using MTT assay. Cells were treated with 100 to 0.097 µg/mL of BSA-PEHA NPs and incubated at 24, 48 and 72 h. [*p* values < 0.05 (*), <0.01 (**)]. (**B**) Cellular uptake study of BSA-PEHA NPs using confocal microscopy. Scale bar: 20 µm. (**C**) Electrophoretic Mobility Shift assay of BSA-PEHA and eGFP mRNA binding. Lane 1: mRNA, Lane 2: BSA-PEHA (25 µg/mL) and 1 µg mRNA, Lane 3: BSA-PEHA (25 µL, 25 µg/mL) and 2 µg mRNA, Lane 4: BSA-PEHA (25 µg/mL) NPs. (**a**) Gel image taken in a UV transilluminator (**b**) Gel after staining with Coomassie blue. pH of the phosphate buffer was maintained at 7.0.

The cellular uptake assay in HD11 cells showed time-dependent internalization of FITC-labeled BSA-PEHA NPs. After 0.5 h, the spherical NPs were observed attaching to the cell membrane (Figure 2B), distinguished using CellMask™ Deep Red Plasma Membrane stain (red channel, 649/666 nm). Hoechst 33,342 stained the nucleus (blue channel, 460/490 nm) (Figure 2B). After 1 h, NPs were internalized and aggregated near the nucleus. By 24 h, most cells contained FITC-labeled NPs, primarily accumulating around the nucleus (Supplementary Figure S1a).

### 3.3. In Vitro Cell Cytotoxicity Study of BSA-PEHA NP Using MTT Assay

The cytotoxicity of BSA-PEHA NPs on HD11 cells was evaluated using an MTT assay. Cells were treated with NP concentrations ranging from 100 µg/mL to 0.0976 µg/mL and incubated for 24, 48, and 72 h. A significant decrease in cell viability (~72%) was observed at 3.125 µg/mL after 24 h, while a dose of 6.25 µg/mL led to ~76% reduction after 48 h (Figure 2A). However, no significant cytotoxicity was detected at 72 h (*p* > 0.05). Further analysis of NP components (BSA, PEHA, and media) showed no cytotoxic effects (Supplementary Figure S2). Flow cytometry findings (Supplementary Figure S5) confirmed a dose-dependent reduction in cell viability from 1 µg/mL to 200 µg/mL, supporting the MTT assay results.

### 3.4. Binding Study of mRNA with BSA-PEHA NPs Using Electrophoretic Mobility Shift Assay (EMSA) and Evaluation of Structure Change Using CD Spectra

**Figure 3:**
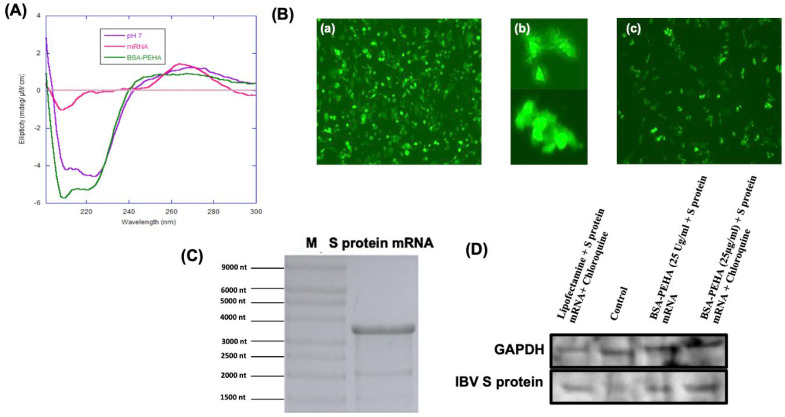
(**A**) CD spectra of BSA-PEHA, mRNA and BSA. (**B**) Expression of eGFP encoding mRNA using Lipofectamine 2000 and BSA-PEHA NPs as the delivery vehicle in HD11 cells. (**a**) Expression of eGFP encoding mRNA with Lipofectamine 2000 (10×). (**b**) Expression of eGFP encoding mRNA with BSA-PEHA (25 µg/mL) (40×). (**c**) Expression of eGFP encoding mRNA BSA-PEHA (25 µg/mL) in presence of chloroquine (25 µM) (10×). (**C**) Denaturing gel electrophoresis of IBV S protein encoding mRNA. (**D**) Western blot image of IBV S protein encoding mRNA (128 kDA) in HD11 cells expressed in presence and absence of chloroquine.

The binding of BSA-PEHA NPs with mRNA was investigated using electrophoretic mobility shift assay (EMSA). BSA-PEHA (25 µL, 25 µg/mL) was conjugated with 1 µg and 2 µg of mRNA at pH 7.0. In the EMSA, free mRNA and free BSA-PEHA NPs showed quick migration toward the anode and cathode, respectively (Figure 2C(a), well 4; Figure 2C(b), well 1). However, when conjugated, the mobility of the complex decreased, indicating successful binding of the BSA-PEHA NPs to the mRNA (lanes 2 and 3). Circular dichroism (CD) spectra confirmed that BSA retained its secondary structure after conversion to BSA-PEHA, with negative bands at 222 nm and 208 nm (Figure 3A). The IVT IBV S protein encoding mRNA displayed a negative band at 210 nm and a positive band at 260 nm. After binding with BSA-PEHA, the conjugate retained the BSA secondary structure, with the addition of a positive band at 260 nm, confirming successful conjugation of mRNA with BSA-PEHA.

### 3.5. Expression of eGFP and IVT IBV S Protein Encoding mRNA in HD11 Cells

The IVT IBV S protein encoding mRNA was prepared in 1 mM sodium citrate buffer (pH 6.4) with a concentration of 0.690 mg/mL and an A260/A280 ratio of 1.48. The mRNA, with 3489 nucleotides (Figure 3C), was stored at −80 °C till further use. HD11 cells were transfected with eGFP encoding mRNA and IVT IBV S protein encoding mRNA using either Lipofectamine 2000 or BSA-PEHA as transfection reagents. Cells transfected with BSA-PEHA showed low expression of both eGFP and IBV S proteins without chloroquine (Figure 3B(a)), with eGFP-expressing cells forming clumps (Figure 3B(b)). To enhance protein expression, cells were incubated with 25 µM chloroquine, which increased endosomal escape. Western blot analysis confirmed that chloroquine significantly boosted the expression of S protein expression (Figure 3D) (Supplementary Figure S6).

#### 3.5.1. Antibody Response to BSA-PEHA Conjugated with IBV S Protein Encoding mRNA

**Figure 4:**
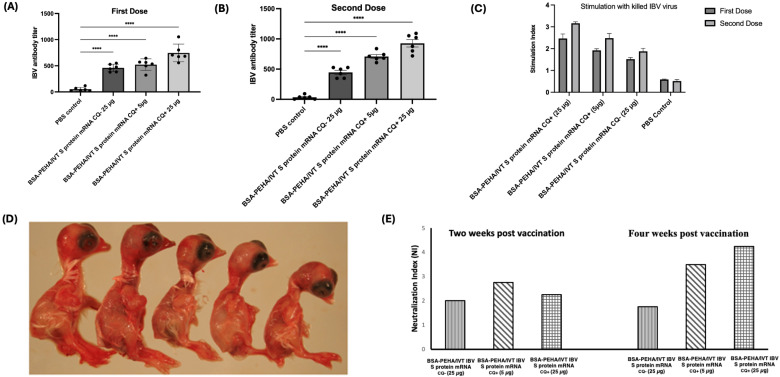
*(***A**) Titer of antibody against IBV S protein in different groups after first (*p* values < 0.0001 (****), and (**B**) second dose. (**C**) PBMC stimulation with killed IBV virus. (**D**) Virus neutralization study in embryonated chicken eggs. When the virus is neutralized, embryos exhibit normal development (left two). In contrast, if the virus remains active, the embryos display stunted growth (right three) (**E**) Serum neutralization assay result. Serum neutralization of chickens immunized with different combinations of BSA-PEHA/IVT IBV S protein encoding mRNA at two and three weeks after vaccination.

To assess the antibody response elicited by BSA-PEHA/IVT IBV S protein encoding mRNA, an ELISA test was performed on serum samples from chickens two and four weeks after receiving the first and second doses of vaccination. Using the IBV ELISA kit from IDEXX, the log10 antibody titers were calculated based on the absorbance readings and the formula: Log10 Titer = 1.09 (log10 S/P) + 3.36, where S/P is the ratio of sample to control. A positive result (titer > 396) indicated exposure to IBV. The results showed a significant increase in antibody levels against IBV S protein in chickens vaccinated with BSA-PEHA/IVT IBV S protein encoding mRNA, regardless of chloroquine addition, and at different mRNA concentrations, compared to the PBS control group (Figure 4A,B). Two weeks after the first dose, the titers for serum from chickens vaccinated with 25 µg of mRNA without chloroquine, 5 µg with chloroquine, and 25 µg with chloroquine were 475.48 ± 72.02, 522.15 ± 110.12, and 745.24 ± 169.86, respectively (Figure 4A). Four weeks after the second dose, the titers were 445.98 ± 84.18, 709.40 ± 77.72, and 927.44 ± 150.68, respectively (Figure 4B). Significant differences were observed between groups with and without chloroquine, with the 25 µg mRNA group showing higher antibody production compared to the 5 µg mRNA group for both doses.

#### 3.5.2. Stimulation of Cell-Mediated Immune Response in PBMCs from Chickens Vaccinated with the BSA-PEHA/IVT IBV S Protein Encoding mRNA

PBMCs from chickens immunized with different combinations of BSA-PEHA/IVT IBV S protein encoding mRNA were stimulated with heat-inactivated IBV virus to assess immune memory activation (14 and 28 day). The proliferation of PBMCs, measured as a change in absorbance, was compared to PBMCs from control chickens inoculated with PBS. The results demonstrated that T-cells were activated in the presence of 10 µg/mL of inactivated IBV virus (Figure 4C). PBMCs from chickens vaccinated with 5 µg and 25 µg of IBV S protein encoding mRNA, with or without chloroquine, showed increased proliferation compared to PBS controls, indicating immune recall. A three-fold increase in the stimulation index was observed when 25 µg of mRNA was administered with chloroquine, and a 1.5-fold increase was noted without chloroquine, compared to the PBS control.

#### 3.5.3. Evaluation of Efficacy of the BSA-PEHA/IVT IBV S Protein Encoding mRNA Using Serum Neutralization Assay

The efficacy of the BSA-PEHA/IVT IBV S protein encoding mRNA vaccine was assessed using a serum neutralization assay in embryonated chicken egg (Figure 4D), with pooled serum from each experimental group. The neutralization index (NI) was calculated, with an NI of ≥1.5 considered positive. Results showed NI values of 4.25, 3.5, and 2.25 for BSA-PEHA/IVT IBV S protein encoding mRNA (25 µg), BSA-PEHA/IVT IBV S protein mRNA (5 µg) with chloroquine, and BSA-PEHA/IVT IBV S protein encoding mRNA without chloroquine, respectively, four weeks post-immunization (Figure 4E). These findings indicate that the vaccine effectively increases antibody titers in chickens, with a twofold increase in NI when 25 µg of mRNA was administered with chloroquine, highlighting the vaccine’s potential to enhance immune responses.

## 4. Discussion

Avian infectious bronchitis (IBV) vaccines have evolved over the years to counter its challenges. Nanoparticles, with diameters under 100 nm, made from various materials like carbon, metal, ceramic, semiconductors, polymers, or lipids, exhibit unique properties useful across many fields [29,30,31]. In our study, we employed a novel BSA-based nanoparticle for delivering IBV S protein encoding mRNA.

Our confocal microscopy results from the cellular uptake study in HD 11 cells show that the NP is internalized by the cell in a time-dependent fashion. After 30 min of incubation, the NPs are found to localize on the plasma membrane’s surface, indicating that there is some initial interaction between the NPs and the cell membrane. The NPs enter the cell by interacting with the components of the plasma membrane and reach the inside of the cell through a process called endocytosis [36]. Although there are several endocytosis mechanisms, surface charge has been shown to play a significant influence in defining the method of uptake and intracellular fate of the NPs. The process of endocytosing positively charged NPs is limited to clathrin-mediated endocytosis [37]. On further incubation, instead of dispersing throughout the cytoplasm, NPs appear to be aggregating close to the nucleus, suggesting that certain cellular targeting or trafficking systems are directing the NPs toward subcellular compartments. This result validates the theory that active interactions and intracellular transport play a role in the cellular uptake of NPs, rather than it being a passive process. Additionally, the NPs’ structure greatly influences the cell’s ability to absorb them. Studies reveal that spherically formed NPs exhibit higher cellular absorption in comparison to other NP shapes [38,39,40,41,42,43]. Furthermore, size also plays an essential role in determining the mechanism of uptake of the NPs. Larger NPs (500 nm) are internalized through caveolae-mediated endocytosis while the smaller ones (<200 nm) are taken up through clathrin-mediated endocytosis [44]. Additionally, smaller-sized NPs are vigorously internalized by cells as compared to the larger ones [45].

In the cell viability assay, the HD11 cells exposed to the NPs demonstrated a dose-dependent suppression of cell proliferation, resulting in a decrease in the cell viability percentage. Exposure to progressively higher concentrations of nanoparticles resulted in a reduction in cell viability. Notable cytotoxicity was observed at 3.125 µg/mL after 24 h and at 6.25 µg/mL following 48 h of treatment, whereas no significant cell death was detected after 72 h. These findings suggest that the toxicity of BSA PEHA NPs to cells increases with their concentration. Moreover, no discernible cytotoxicity was seen in control trials including the individual NP components, PEHA and BSA, indicating that the effects seen are unique to the NPs themselves. Our results further emphasize how crucial it is to consider elements like NP composition, size, shape, and surface charge when determining how lethal the particles are. Our results imply that smaller-sized NPs may exhibit increased toxicity, presumably due to enhanced cellular absorption or interactions with intracellular environments, even if the precise processes behind NP-induced cytotoxicity are still not fully understood [46]. Previous studies showing a link between NP size and cytotoxicity support this theory [47,48,49,50,51,52,53,54]. Furthermore, flow cytometry analysis revealed a clear concentration-dependent decrease in cell viability with escalating NP levels, supporting the MTT assay findings.

EMSA is a swift and sensitive method to determine the interaction between protein and nucleic acid based on the principle that when there is binding between the protein and the nucleic acid the mobility decreases as compared to the free nucleic acid [55]. In this study, we were able to ascertain that there is binding that occurred between the cationic BSA-PEHA and mRNA. The negatively charged nucleic acids are impacted by the interactions between the positively charged polyamine of BSA-PEHA. The intricate relationships between polyamines and nucleic acids have been extensively studied [56,57,58,59,60,61,62,63,64,65,66,67,68], although the precise mechanisms are still unknown. Electrostatic interactions between polyamines and nucleic acid targeting both major and minor groove and intra and interstrand interactions [68] have all been postulated but the thermodynamics of their binding to nucleic is still not fully understood.

mRNA has gained substantial interest in the vaccine field after the COVID-19 vaccine was introduced. Avian infectious bronchitis (IBV) vaccines have evolved over the years to counter their challenges. Initially, live attenuated vaccines were introduced in the 1950s, generated by passing the virus through chicken embryos. However, concerns over reversion to virulence and interference with maternal antibodies [69] prompted the development of inactivated oil-emulsion vaccines [70,71]. In our study, we employed a novel BSA-based NP for delivering IBV S protein-encoding mRNA. mRNA molecules are efficient in translating the message from DNA to the desired protein. In this study, we designed an IVT mRNA by incorporating changes such as adding cap 1 analog. This modification mimics the naturally occurring mRNA cap structure, facilitating efficient translation initiation and preventing mRNA degradation [72]. Its structure consists of 5ʹ and 3ʹ untranslated regions (UTRs) and open reading frame (ORF), also called the coding region and the poly(A) tail. Furthermore, 5′ and 3′ UTRs assist in the recognition of the transcripts by the ribosomes [73]. The polyadenylation with 110 poly (A) tail provides stability to the mRNA molecule and is crucial for efficient mRNA translation and stability in eukaryotic cells. The optimum length for polyadenylation is 100 to 120 nucleotides [74]. The ORF region has coding sequences for the spike protein of the IBV virus. Finally, codon optimization was performed for the sequence due to the challenge of redundancy in the genetic code. The main objective of codon optimization is to enhance the expression of the target protein and also help in the stability of the mRNA molecule [75].

To investigate the successful expression of mRNA by BSA-PEHA NPs we employed the eGFP encoding mRNA. For control, we utilized the common transfecting agent Lipofectamine 2000. This is a cationic liposome-based reagent and has high transfection efficiency [76]; it has been successfully used to transfect short-interfering RNAs into mammalian cells [77,78]. We observed that the IVT IBV S protein encoding mRNA was successfully expressed with both Lipofectamine 2000 and BSA-PEHA in HD11 cells. The expression was further enhanced in the presence of chloroquine. One of the proposed mechanisms by which chloroquine helps in endosomal escape is that it efficiently enters the cell because of being a weak base [79]. As a result, it becomes protonated and increases the basicity of the endosomes thus causing the efflux of drugs from the cytosol. Another mechanism is the increase in osmotic pressure. This leads to leakage of water into the endosomes leading to rupture [79]. These promising results demonstrate that the BSA-PEHA in combination with chloroquine can improve the mRNA delivery.

The ELISA assay revealed a significant increase in antibody titer against IBV S protein in chickens vaccinated with BSA-PEHA/IVT IBV S protein encoding mRNA vaccine compared to the control group. The antibody titers were higher in chickens inoculated with higher doses of mRNA along with chloroquine. This suggests that both the dosage of the mRNA and co-administration of chloroquine play crucial roles in enhancing the antibody response. Additionally, the evaluation of the vaccine’s efficacy through a serum neutralization assay further corroborates its potential in enhancing immune response. The observed neutralization indices, particularly the twofold increase observed upon boosting with 25 µg of mRNA in the presence of chloroquine, highlight the vaccine’s potency in eliciting a protective immune response against IBV. The stimulation of cell-mediated immune response in PBMCs from vaccinated chickens further supports the efficacy of the vaccine. Importantly, PBMCs from chickens vaccinated with BSA-PEHA/IVT IBV S protein encoding mRNA exhibited higher proliferation when exposed to heat-inactivated IBV virus compared to controls, highlighting the capability of immune recall. Thus, based on these observations, cationic BSA NP presents a promising vaccine strategy for developing mRNA vaccines against various poultry viruses due to its safety, ease of production, cost-effectiveness, and high stability.

## 5. Conclusions

This study highlights the potential of cationic BSA-polyamine NPs as an effective delivery system for mRNA-based poultry vaccines. This approach addresses key challenges in vaccine development by facilitating efficient cellular uptake, mRNA protection, and enhancing expression with chloroquine. The strong immune response induced by the BSA-PEHA/IVT IBV S protein encoding mRNA vaccine demonstrates its promise for improving poultry disease control, particularly against IBV. This strategy offers a safer, cost-effective, and scalable solution for advancing next-generation vaccines in poultry health.

## Data Availability

The data can be shared up on request.

## Supplementary Materials

The following supporting information can be downloaded at https://www.mdpi.com/article/10.3390/vaccines13060568/s1, Figure S1: Confocal microscopy image of BSA-PEHA NPs and gel electrophoresis image of the NPs, Figure S2: Cell viability assay for media, BSA and PEHA control, Figure S3: Cell viability of chloroquine treated HD11 cells using MTT assay, Figure S4: S protein sequence used for IVT mRNA design. Figure S5: Quantification of cell cytotoxicity to BSA-PEHA NPs using Flow cytometry by staining cells using a Viability/Cytotoxicity Assay Kit for live and dead cells; Figure S6: Densitometry analysis of Western blot image of the S protein after in vitro expression in HD11 cells.
