# Supplementary material for: Nanoparticle-Based mRNA Vaccine Induces Protective Neutralizing Antibodies Against Infectious Bronchitis Virus in In-Vivo Infection
Source: Vaccines (Basel). 2025 May 26;13(6):568. doi: 10.3390/vaccines13060568 (PMC12197329; doi:10.3390/vaccines13060568)
Supplement: Supplementary file 1 [file vaccines-13-00568-s001.zip › vaccines-3605671-supplementary.pdf]

vaccines

MDPI

Supplementary figures:

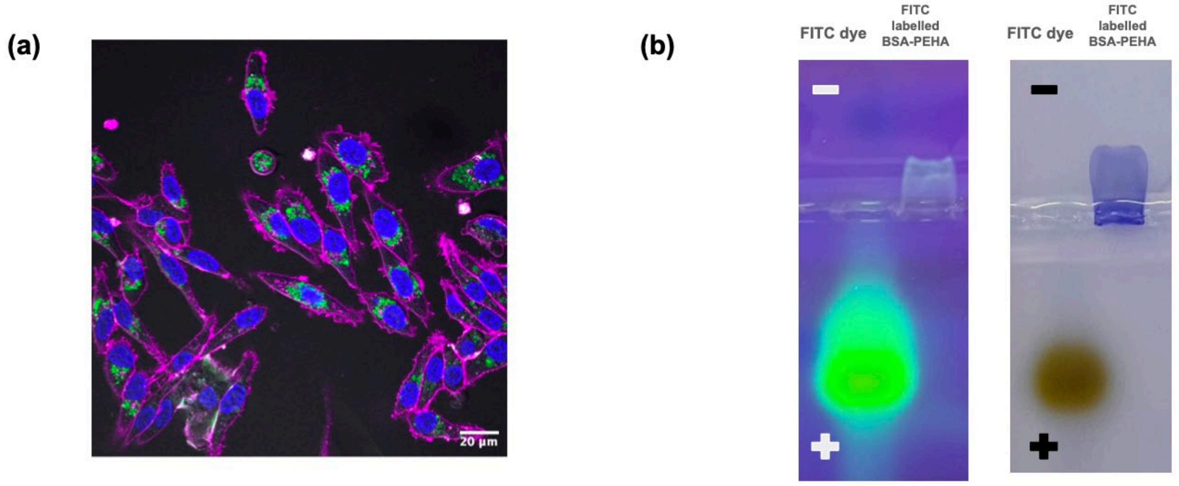

Figure S1: Confocal microscopy image of BSA-PEHA NPs and gel electrophoresis image of the NPs. (a) Confocal image of FITC labeled BSA-PEHA NPs treated HD11 cells after 24 hours of incubation (Scale: 20μm). (b) Gel image showing the FITC labeled BSA-PEHA and FITC dye after UV exposure and Coomassie blue stain, respectively.

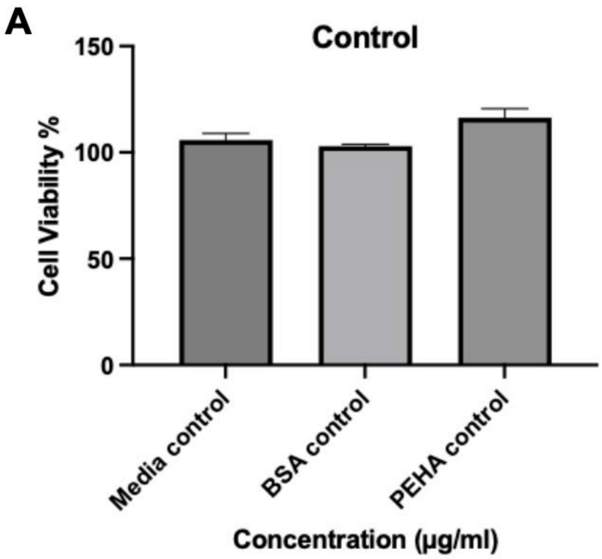

Figure S2. Cell viability assay for media, BSA, and PEHA control Cytotoxicity analysis of chloroquine was done to evaluate the cytotoxicity of chloroquine on HD11 cells. The HD11 cells were grown overnight and then treated with different concentrations of chloroquine. The percentage of viable cells was measured after 24 and 48 hours using the MTT assay. A significant decrease in cell viability was observed at a concentration of 125 μM at 24 hours and 31.25 μM at 48 hours of incubation. The results indicated that the chloroquine exhibited cytotoxicity after 48 hrs and the cytotoxicity was dose dependent. The chloroquine shows cytotoxicity after 48 hrs when the concentration was increased to 30 μM and it was found to decrease the cell proliferation in a dose-dependent manner.

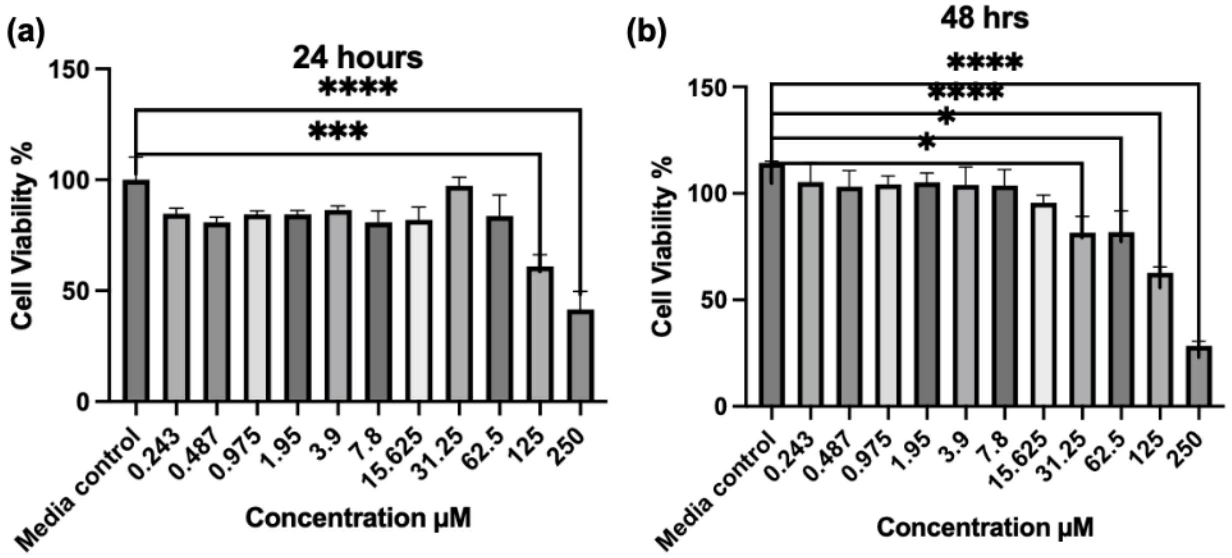

Figure S3. Cell viability of chloroquine-treated HD11 cells using MTT assay. Cells were treated with 250 to 0.243 µM of chloroquine and incubated at 24 (a) and 48 (b) hours. The data was obtained by using GraphPad Prism v10 software. Data points represent n = 3 experiments with triplicates, for each experiment ± standard deviation. *P<0.05, ** P< 0.01 and *** P<0.001.

atgttg gtaacacctc ttttactagt gactcttttg tgtgtactat gtagtgctgc tttgtatgac agtagttctt acgtttacta ctaccaaagt gcctttagac
cacctaatgg ttggcattta cacgggggtg cttatgcggt agttaatatt tctagcgaat ctaataatgc aggctcttca cctgggtgta ttgttggtac tattcatggt
ggtcgtgttg ttaatgcttc ttctatagct atgacggcac cgtcatcagg tatggcttgg tctagcagtc agttttgtac tgcacactgt aacttttcag atactacagt
gtttgttaca cattgttata aatatgatgg gtgtcctata actggcatgc ttcaaaagaa tttttacgt gtttctgcta tgaaaaatgg ccagcttttc tataatttaa
cagttagtgt agctaagtac cctactttta aatcatttca gtgtgttaat aatttaacat ccgtatattt aaatggtgat cttgtttaca cctctaatga gaccacagat
gttacatctg caggtgttta ttttaaagct ggtggaccta taacttataa agttatgaga gaagttaaag ccctggctta ttttgttaat ggtactgcac aagatgttat
tttgtgtgat ggatcaccta gaggcttgtt agcatgccag tataatactg gcaatttttc agatggcttt tatcctttta ttaatagtag tttagttaag cagaagttta
ttgtctatcg tgaaaatagt gttaatacta cttttacgtt acacaatttc acttttcata atgagactgg cgccaaccct aatcctagtg gtgttcagaa tattcaaact
taccaaacac aaacagctca gagtggttat tataatttta atttttcctt tctgagtagt tttgtttata aggagtctaa ttttatgtat ggatcttatc acccaagttg
taattttaga ctagaaacta ttaataatgg cttgtggttt aattcacttt cagtttcaat tgcttacggt cctcttcaag gtggttgcaa gcaatctgtc tttagtggta
gagcaacttg ttgttatgct tattcatatg gaggtccttc gctgtgtaaa ggtgtttatt caggtgagtt agatcttaat tttgaatgtg gactgttagt ttatgttact
aagagcggtg gctctcgtat acaaacagcc actgaaccgc cagttataac tcgacacaat tataataata ttactttaaa tacttgtgtt gattataata tatatggcag
aactggccaa ggttttatta ctaatgtaac cgactcagct gttagttata attatctagc agacgcaggt ttggctattt tagatacatc tggttccata gacatctttg
ttgtacaagg tgaatatggt cttacttatt ataaggttaa cccttgcgaa gatgtcaacc agcagtttgt agtttctggt ggtaaattag taggtattct tacttcacgt
aatgagactg gttctcagct tcttgagaac cagttttaca ttaaaatcac taatggaaca cgtcgtttta gacgttctat tactgaaaat gttgcaaatt gcccttatgt
tagttatggt aagttttgta taaaacctga tggttcaatt gccacaatag taccaaaaca attggaacag tttgtggcac ctttacttaa tgttactgaa aatgtgctca
tacctaacag ttttaattta actgttacag atgagtacat acaaacgcgt atggataagg tccaaattaa ttgtctgcag tatgtttgtg gcaattctct ggattgtaga
gatttgtttc aacaatatgg tcctgtttgt gacaacatat tgtctgtagt aaatagtatt ggtcaaaaag aagatatgga acttttgaat ttctattctt ctactaaacc
ggctggtttt aatacaccat ttcttagtaa tgttagcact ggtgagttta atatttctct tctgttaaca actcctagta gtcctagaag gcgttctttt attgaagacc
ttctatttac aagcgttgaa tctgttggat taccaacaga tgacgcatac aaaaattgca ctgcaggacc tttaggtttt cttaaggacc ttgcgtgtgc tcgtgaatat
aatggtttgc ttgtgttgcc tcccattata acagcagaaa tgcaaacttt gtatactagt tctctagtag cttctatggc ttttggtggt attactgcag ctggtgctat
accttttgcc acacaactgc aggctagaat taatcacttg ggtattaccc agtcactttt gttgaagaat caagaaaaaa ttgctgcttc ctttaataag gccattggtc
gtatgcagga aggttttaga agtacatctc tagcattaca acaaattcaa catgttgtta ataagcagaa tgctattctt actgagacta tggcatcact taataaaaat
tttggtgcta tttcttctct gattcaagaa atctaccagc aacttgacgc catacaagca aatgctcaag tggatcgtct tataactggt agattgtcat cactttctgt
tttagcatct gctaagcagg cggagcatat tagagtgtca caacagcgtg agttagctac tcagaaaatt aatgagtgtg ttaagtcaca gtctattagg tactcctttt
gtggtaatgg acgacatgtt ctaaccatac cgcaaaatgc acctaatggt atagtgttta tacacttttc ttatactcca gatagttttg ttaatgttac tgcaatagtg
ggtttttgtg taaagccagc taatgctagt cagtatgcaa tagtacctgc taatggtagg ggtattttta tacaagttaa tggtagttac tacatcacag cacgagatat
gtatatgcca agagctatta ctgcaggaga tatagttacg cttacttctt gtcaagcaaa ttatgtaagt gtaaataaga ccgtcattac tacattcgta gacaatgatg
atttgattt taatgacgaa ttgtcaaaat ggtggaatga cactaagcat gagctaccag actttgacaa attcaattac acagtaccta tacttgacat tgatagtgaa
attgatcgta ttcaaggcgt tatacagggt cttaatgact ctttaataga ccttgaaaaa ctttcaatac tcaaaactta tattaagtgg ccttggtatg tgtggttagc
catagctttt gccactatta tcttcatctt aatactagga tgggttttct tcatgactgg atgttgtggt tgttgttgtg gatgctttgg cattatgcct ctaatgagta
agtgtggtaa gaaatcttct tattacacga cttttgataa cgatgtggta actgaacaaa acagacctaa aaagtctgtt **taa**

Figure S4. S protein sequence used for IVT mRNA design.

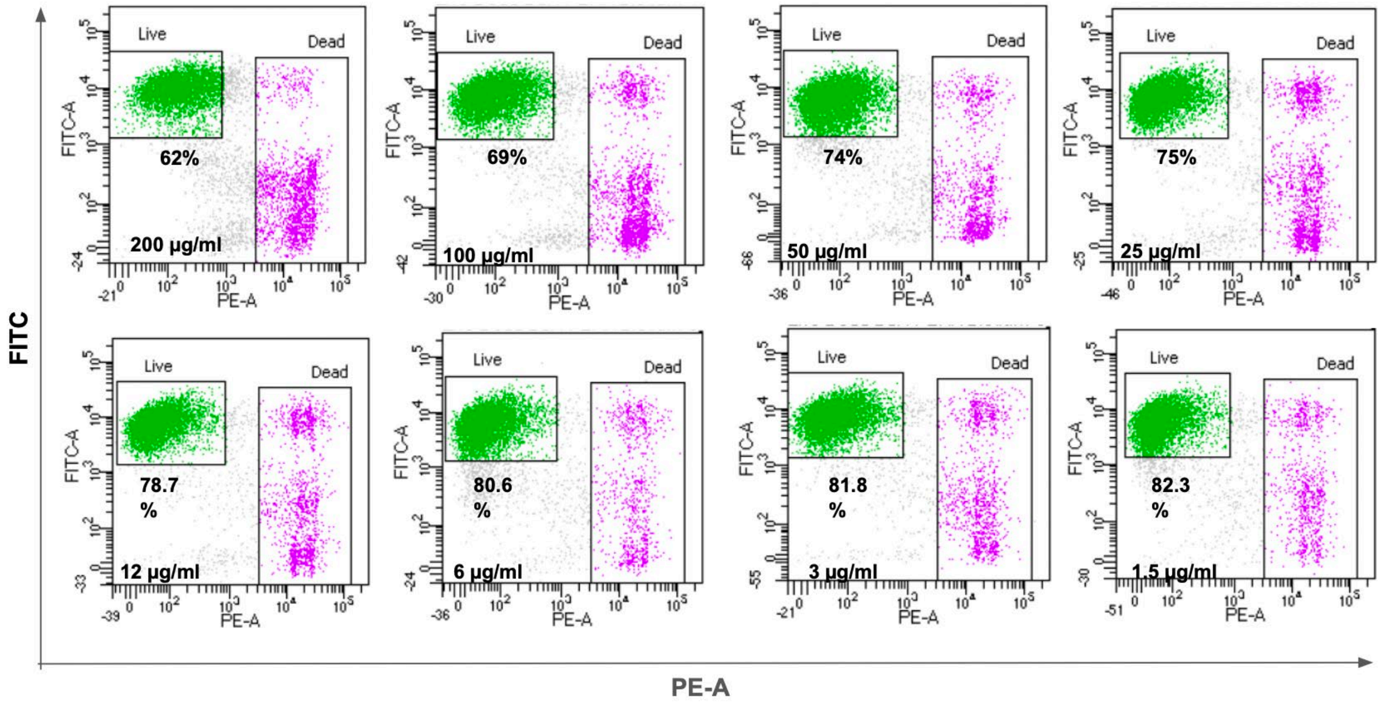

Figure S 5: Quantification of cell cytotoxicity to BSA-PEHA NPs using Flow cytometry by staining cells using a Viability/Cytotoxicity Assay Kit for live and dead cells.

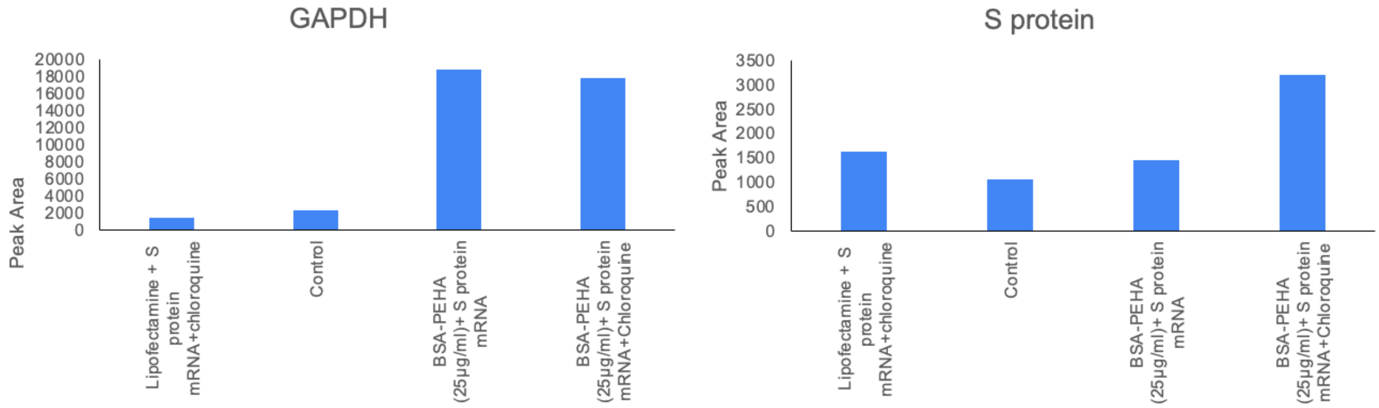

Figure S6: Densitometry analysis of Western blot image of the S protein after *in vitro* expression in HD11 cells.
